# Koumine’s Therapeutic Impact on Hepatocellular Carcinoma: A Combined Network Pharmacology and Experimental Study

**DOI:** 10.3390/biomedicines14061250

**Published:** 2026-05-30

**Authors:** Hailing Lin, Yuli Tang, Lingfei Shi, Shengjie Zhu, Wenqiang Yan, Weihong Chen, Wancai Que

**Affiliations:** 1 Department of Pharmacy, Fujian Medical University Union Hospital, 29 Xin Quan Rd, Gulou, Fuzhou 350001, China; lhailing@fjmu.edu.cn (H.L.); l1ngfei@163.com (L.S.); zhusj215@hotmail.com (S.Z.); ywq890828@163.com (W.Y.); 2 Phase I Clinical Trial Unit, Fujian Medical University Union Hospital, 29 Xin Quan Rd, Gulou, Fuzhou 350001, China; yuriitang@163.com; 3 Department of Anesthesiology, The Second Affiliated Hospital, Fujian Medical University, Quanzhou 362000, China

**Keywords:** Koumine, hepatocellular carcinoma, network pharmacology, mechanism of action, molecular docking, in vitro/in vivo experiments

## Abstract

**Background:** Koumine is a bioactive alkaloid derived from the traditional medicinal plant *Gelsemium elegans*. Although it has demonstrated anti-tumor effects in various cancers, its specific role and mechanism in hepatocellular carcinoma (HCC) remain unclear. This study aims to investigate the anti-HCC effects of Koumine and elucidate the underlying molecular mechanisms. **Methods:** A network pharmacology approach was employed to predict potential targets and pathways of Koumine against HCC. The binding affinities between Koumine and core targets were validated using molecular docking. In vitro, the effects of Koumine on the proliferation, migration, and invasion of HCC cells were assessed, and the expression levels of key proteins were examined. In vivo, the anti-tumor efficacy and toxicity of Koumine were evaluated using a murine xenograft model. **Results:** Network pharmacology analysis identified 124 potential targets of Koumine against HCC, with 10 core targets (e.g., P38, JAK1, JAK2, GRB2) and key pathways involving MAP2K1, P38, JAK1, and MET being implicated. Molecular docking confirmed strong binding affinities between Koumine and these core targets. In vitro experiments demonstrated that Koumine dose-dependently inhibited the proliferation, migration, and invasion of HCC cells and modulated the expression and phosphorylation of P38. In vivo results showed that Koumine significantly suppressed tumor growth without causing notable toxicity. **Conclusions:** This study systematically reveals that Koumine exerts its anti-HCC effects by targeting the MAP2K1, P38, JAK1, JAK2, and MET signaling pathways. These findings highlight the potential of Koumine as a novel and safe therapeutic agent for the treatment of hepatocellular carcinoma.

## 1. Introduction

Hepatocellular carcinoma (HCC) is a leading global malignancy [1]. In 2020, the incidence rate of liver cancer was the sixth among all cancers, approximating 906,000 new cases, while its mortality rate stood third with 830,000 deaths, nearly half of the occurrence in China [2]. Representing 70–85% of liver cancers, HCC underscores the urgent need for effective treatments [3]. For advanced stages, options are limited, with a poor prognosis: the median survival is less than one year, and the 5-year survival rate is below 9% [4]. Given that 50% of diagnoses are already at an advanced stage, systemic treatments are the cornerstone. However, targeting drugs, for example, sorafenib, lenvatinib, and regorafenib have displayed unsatisfactory effects and considerable side effects, such as diarrhea, rash, hypertension, and fatigue, complicating treatment owing to resistance issues [5,6,7,8]. Lately, monoclonal antibodies targeting PD-1/PD-L1 have emerged as promising but variably effective treatments, with side effects like immune myocarditis and hepatitis [9]. Thus, developing new therapies to improve survival and quality of life remains critically important.

*Gelsemium elegans* Benth (GEB), as a Loganiaceae family member, is rich in indole alkaloids [10] and is recognized for its diverse pharmacological activities, such as anti-tumor, anti-inflammatory, and immunomodulatory effects [11]. Gao et al. [12] have demonstrated that GEB alkaloid monomers significantly suppressed the proliferation of HepG2 cells in vitro, with minimal cytotoxicity. This suppression was mediated by the alterations in cell cycle progression and the activation of Caspase-8, Caspase-9, and Caspase-3, culminating in anti-tumor effects. Wang [13] found that Koumine (KM), a principal monomer in GEB, impeded cell growth of colorectal cancer by downregulating B-cell lymphoma/leukemia-2 gene (Bcl-2) expression and triggering apoptosis, with concomitant increased levels of Bax, cytochrome oxidase (CytC), and Caspase-3. A previous study [14] observed potent cytotoxicity of GEB alkaloids against human lung adenocarcinoma cells (A549), and another study [15] reported similar effects on human epidermoid carcinoma cells (A431), highlighting Koumine’s role. Despite people having recognized the pharmacological potential of Koumine, research on its mechanisms of anti-HCC is scant. Previous studies have primarily focused on the cytotoxic effects of Gelsemium alkaloids in colorectal, lung, or epidermoid carcinoma cells, with limited exploration in HCC and a notable absence of systematic target deconvolution. Therefore, our research utilizes network pharmacology, molecular docking, and experimental investigations to unravel Koumine’s mechanism of action against HCC and the efficacy of Koumine. This study distinguishes itself from prior work by (1) establishing a comprehensive ‘compound-target-pathway-disease’ network specific to HCC, (2) validating direct target engagement of P38 via CETSA, and (3) correlating in vitro proteomics with in silico predictions to provide a multi-omics mechanistic framework for Koumine’s anti-HCC activity. In conclusion, our study provides new theoretical basic and experimental support for the treatment of HCC with Koumine, potentially guiding future liver cancer treatments.

## 2. Materials and Methods

### 2.1. Koumine

Koumine was purchased from Chengdu Alfa Biotechnology Co., Ltd. (Chengdu, China). The molecular formula of the Koumine is C20H22N2O. The purity is 99.42%. The chemical structure of Koumine was shown in Table 1. An HPLC-based chemoprofile was shown in Table 2.

### 2.2. Cell Culture and Reagents

Hepatocellular carcinoma cell lines, HepG2 and Huh7, were from the American Type Culture Collection (ATCC, Manassas, VA, USA). They were maintained in DMEM (11875101, Gibco, Grand Island, NY, USA) with 10% fetal bovine serum (10099141C, Gibco, Grand Island, NY, USA). Oxaliplatin (L-OHP) injections from Jiangsu Hengrui Medicine Co., Ltd. (Lianyungang, China).

### 2.3. Cell Proliferation Experiment

HepG2 and Huh7 cells were seeded in 96-well plates at 7500 cells per well (100 μL medium per well). Following overnight incubation, cells were treated with increasing concentrations of Koumine (0, 10, 100, 200, 400, 800, 1600 μg/mL) or L-OHP (0, 1, 2, 4, 8, 16, 32 μg/mL) as a positive control. Control wells included drug-free cells and blank medium, all performed in six replicates. After 48 h, cell viability was assessed using the CCK-8 assay, and absorbance was measured at 450 nm. The experiment was conducted in triplicate.

### 2.4. Cell Scratch Healing Experiment

Initially, 3000 cells per well were seeded in a 6-well plate and allowed to form a monolayer. After 12 h, a scratch was created using a sterile pipette tip. The medium was then replaced with either a solvent control, varying concentrations of Koumine, or L-OHP. For HepG2 cells, treatments included control (medium), Koumine (KM) (400 μg/mL), and L-OHP (6 μg/mL). For Huh7 cells, treatments included control, KM (400 μg/mL), and L-OHP (8 μg/mL). Wound closure was monitored microscopically at intervals over 48 h. The wound area was quantified using ImageJ software (version 1.54f) (Wound Healing Size Tool). For each well, measurements were taken at three distinct, predefined positions along the scratch. The average wound width at each time point was normalized to the average initial (0 h) width of the same well, and the percentage of wound closure relative to the control group was calculated to evaluate cell migration.

### 2.5. Colony Formation Assay

Colony Formation Assay 400 cells were seeded per well in a 6-well plate and allowed to attach for 24 h. The groups were: for HepG2, control, KM (400 μg/mL), and L-OHP (6 μg/mL); for Huh7, control, KM (400 μg/mL), and L-OHP (8 μg/mL). Each group had three replicates. Cells were incubated for 7–9 days at 37 °C and 5% CO_2_. Colonies were fixed and stained with 1% formaldehyde and 0.1% crystal violet in PBS, then photographed for analysis.

### 2.6. Transwell Assay

To assess cell migration capacity (without Matrigel), logarithmic growth phase HCC cells were diluted to 2 × 10^5^ cells/mL. 300 μL of cell suspension was added to the upper Transwell chamber, with 400 μL of medium in the lower chamber. After 24 h at 37 °C, cells that migrated to the bottom were fixed with absolute ethanol, stained with crystal violet, and photographed. The average number of cells across at least six fields was counted.

### 2.7. Flow Cytometry

Flow Cytometry HCC cells were cultured in 6-well plates (4.0–5.0 × 10^5^ cells/well). The groups were: for HepG2, control, KM (400 μg/mL), and L-OHP (6 μg/mL); for Huh7, control, KM (400 μg/mL), and L-OHP (8 μg/mL). After 24 h, cells were harvested. For cell cycle analysis, cells were stained with DNA staining solution. Apoptosis was assessed using an Annexin V-FITC and PI kit (Multisciences, Hangzhou, China). Analyses were performed on a FACS Fortessa (BD Biosciences, San Jose, CA, USA) and processed with FlowJo software (version 10.8.1, BD Biosciences).

### 2.8. Immunoblot Analysis

Immunoblot Analysis Total protein was isolated using RIPA lysis buffer. Nuclear and cytoplasmic proteins were extracted per the supplier’s protocol (R0050, Solarbio, Beijing, China). For in vitro assays, total protein was isolated from cultured HepG2 and Huh7 cells treated with indicated concentrations of KM (0, 200, 400, 600 µg/mL) for 24 h using RIPA lysis buffer. For in vivo validation, protein was extracted from snap-frozen tumor tissues. All experiments were performed with at least three independent biological replicates. Protein concentrations were quantified by BCA assay. Proteins were resolved by SDS-PAGE, transferred to PVDF membranes, and probed with primary and secondary antibodies. Bands were visualized with ECL and quantified using Image J (version 1.54f).

### 2.9. Cellular Thermal Shift Assay (CETSA)

HepG2 and Huh7 cells were trypsinized, washed, and resuspended in PBS with protease inhibitors (5000 cells/μL). Aliquots (50 μL) were incubated with Koumine (400 μg/mL) or vehicle at 37 °C for 20 min. Samples were heated at a temperature gradient (43–61 °C) for 4 min, then lysed with an equal volume of ice-cold lysis buffer. After centrifugation (13,000× *g*, 3 min, 4 °C), supernatants were analyzed by Western blot for P38 protein stability.

### 2.10. Animal Experiment

Animal Experiment Forty male Nude mice (15–20 g, 5–6 weeks) were from Guangdong Yaokang Biotechnology Co., Ltd. (Guangzhou, China) (Licence No.: SCXK (Yue) 2022-0062; Animal Batch No.: 2022-06DM081). A liver cancer xenograft model was established by subcutaneously injecting 1 × 10^6^ liver cancer cells. When tumors reached a sufficient size, mice were randomized into five groups (*n* = 8/group): NC (control), L-OHP (1 mg/kg), L-KM (1 mg/kg), M-KM (4 mg/kg), and H-KM (8 mg/kg). For one batch (n = 4/group), intraperitoneal injections were given daily for 19 days, with weight and tumor volume recorded every four days. Tumor volume = 0.5 × length × width^2^. After 40 days, four mice from each group were randomly selected and euthanized for tumor excision, imaging, weighing, and histopathological analysis. The remaining four mice per group continued to receive the same treatment and were monitored daily for survival until the predefined endpoint of the study. All procedures were approved by the Animal Research Ethics Committee of Fujian Medical University (IACUCFJMU 2022-0036).

### 2.11. Histopathology and Immunohistochemistry

Histopathology and immunohistochemistry Tumor samples were fixed in 4% paraformaldehyde, paraffin-embedded, and sectioned (4 μm). Sections were stained with hematoxylin and eosin (G1005, Servicebio, Wuhan, China), Masson’s trichrome (G1006, Servicebio, Wuhan, China), and TUNEL (40307, YEASEN, Shanghai, China). Immunohistochemistry was performed with primary antibody Ki67 (AB2008, Beyotime, Shanghai, China, 1:200) and HRP-labeled secondary antibody (A0208, Beyotime, Shanghai, China, 1:1000).

### 2.12. Pharmacokinetic Analysis

A total of 66 Balb/c mice were randomly divided into two groups, with 33 mice in each group. The mice were administered KM intraperitoneally at doses of 1 mg/kg and 8 mg/kg, respectively. At each time point (0, 0.5, 1, 1.5, 2, 4, 6, 8, 12, 16, and 24 h) after administration, 3 mice were selected. The mice were placed in a desiccator, and approximately 10 mL of ether was placed in the lower part of the desiccator. After about 1 min, the mice entered an anesthetic state. Following anesthesia, 200 μL of blood was collected by enucleation. Standard curves were drawn using KM standards of different concentrations through liquid chromatography, and the KM concentration levels in each plasma sample were measured.

### 2.13. Protein Extraction and Analysis by LC–MS/MS

Tumor tissue samples from the murine xenograft model (*n* = 3 per group) were homogenized and sent for proteomics analysis. Protein extraction and analysis by LC–MS/MS Samples were sent for proteomics analysis. Raw data were analyzed using MaxQuant (v1.6.14). Proteins were considered differentially expressed if Fc > 2 or <0.5 and *p* < 0.05. KEGG enrichment analysis was performed on these proteins.

### 2.14. Network Pharmacology for Target Pathways of Koumine in Anti-HCC [16]

Network Pharmacology for Target Pathways of Koumine in Anti-HCC Koumine’s data were from PubChem (refer to Table 1). Potential targets were predicted using TargetNet (http://targetnet.scbdd.com/, probability > 0, accessed on 12 November 2021), PharmMapper (http://www.lilab-ecust.cn/pharmmapper/, zscore > 0, accessed on 12 November 2021), and SwissTargetPrediction (http://www.swisstargetprediction.ch/, probability > 0, accessed on 12 November 2021). HCC-related disease targets were collected from the GeneCards database (https://www.genecards.org/, accessed on 12 November 2021) using the keyword ‘Hepatocellular carcinoma’, filtering for relevance score > 5. The overlapping targets between Koumine and HCC were identified using a Venn diagram. The complete list of predicted targets and overlapping genes is provided in Supplementary File S1–S3.

### 2.15. Protein–Protein Interaction (PPI) Network Topological Analysis

Protein–Protein Interaction (PPI) Network Topological Analysis Common targets were identified with a Venn diagram. A PPI network was constructed in the String database (*Homo sapiens*, confidence > 0.7) and visualized in Cytoscape 3.7.2. The top 10 targets were chosen based on ‘Degree’ scores from the cytohubba plugin.

### 2.16. Functional Enrichment Analysis

Functional enrichment analysis Gene Ontology (GO) and KEGG pathway analyses were conducted using the DAVID database (FDR < 0.01) to understand the biological functions and pathways of key targets. Results were visualized using R software (version 4.0.3).

### 2.17. Construction of Compound-Target-Pathway-Disease Network

Construction of Compound-Target-Pathway-Disease Network A network showing interactions among the compound, targets, pathways, and disease was developed using Cytoscape. Network topology features (‘Degree’, ‘Betweenness’, ‘Closeness’) were analyzed.

### 2.18. Compound-Target Molecular Docking

Compound-Target Molecular Docking 3D structures of compounds (SDF) and proteins (PDB) were from PubChem and PDB, respectively. Structures were prepared in PyMOL 2.4.1. Docking was performed using AutoDock Vina 1.1.2 to analyze interactions and binding energy.

### 2.19. Statistic Analysis

Each experiment was verified with at least three independent replicates. The results were analyzed using GraphPad Prism 10 and presented as mean ± SD. Outliers were identified and excluded using GraphPad Prism. Statistical comparisons were performed using one-way analysis of variance (ANOVA) followed by Tukey’s post hoc test, as well as two-tailed *t*-tests with Bonferroni correction, all conducted using GraphPad Prism 9. Significance levels are indicated as * *p* < 0.05, ** *p* < 0.01, *** *p* < 0.001, **** *p* < 0.0001; n.s. denotes not significant.

## 3. Results

### 3.1. In Vitro Experiments Confirm the Anti-Tumor Effect of Koumine

**Figure 1:**
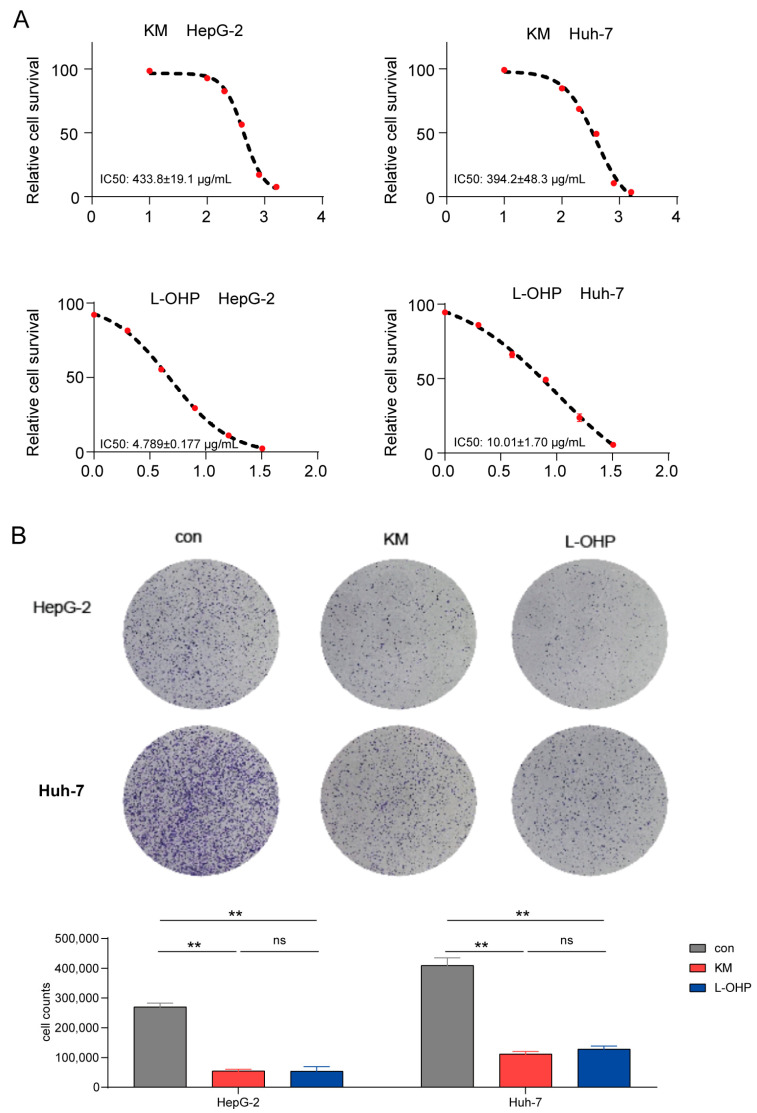
Koumine inhibited the proliferation of HCC cells. (**A**) CCK-8 assay was used to detect the proliferation of HCC cells. (**B**) The colony number of KM (400 μg/mL) and L-OHP (HepG2 6 μg/mL; Huh7 8 μg/mL) group was significantly reduced compared with the control group. The cell counts of HepG2 and Huh7 cells in KM and L-OHP groups were significantly lesser than those in the control group. Data represent three independent experiments. Error bars, mean ± SD. ns, No significant difference. ** *p* < 0.01.

**Figure 2:**
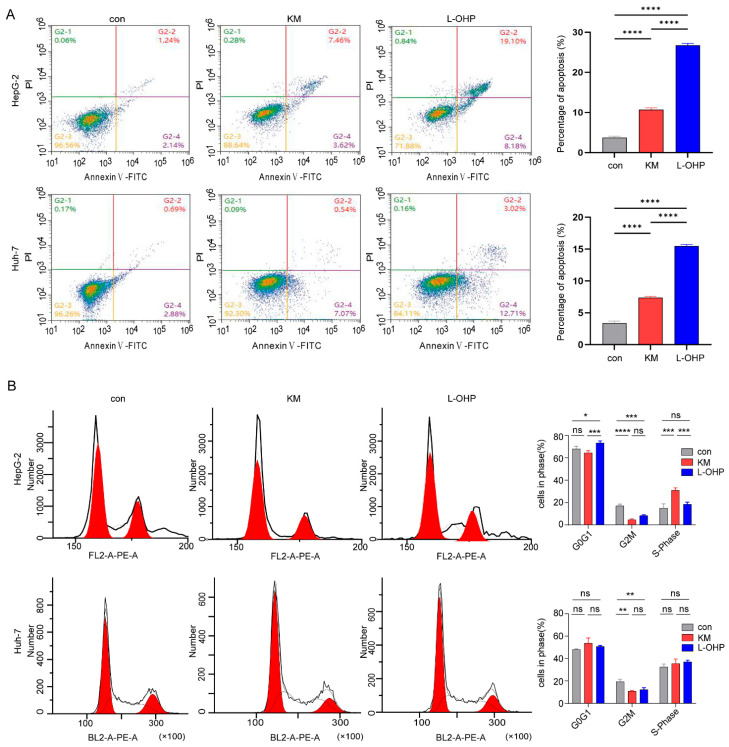
Koumine promoted cell apoptosis and induced cell cycle arrest of HCC cells. (**A**) The apoptosis of HCC cells was detected by flow cytometry. (**B**) The cell cycle of HCC cells was detected by flow cytometry. Data represent three independent experiments. Error bars, mean ± SD. ns, No significant difference. * *p* < 0.05, ** *p* < 0.01, *** *p* < 0.001, **** *p* < 0.0001.

**Figure 3:**
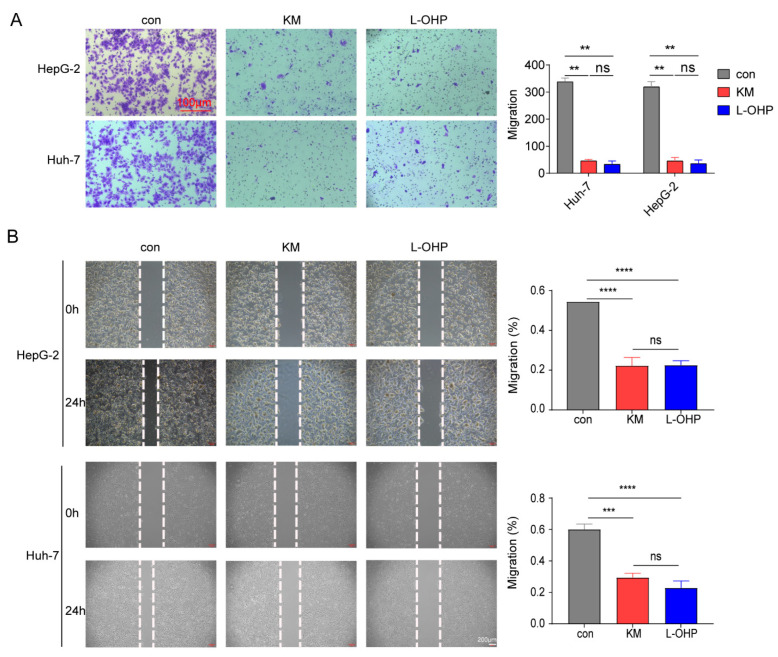
Koumine inhibited cell migration of HCC cells. (**A**) The number of transwell cells in the KM and L-OHP group was significantly lower than that in the control group. (**B**) The scratch healing rate of the KM and L-OHP group was lower than that of the control group. Data represent three independent experiments. Error bars, mean ± SD. ns, No significant difference. ** *p* < 0.01, *** *p* < 0.001, **** *p* < 0.0001.

Oxaliplatin (L-OHP), a new platinum derivative, is the third generation of platinum antineoplastic drugs, which inhibits DNA replication and transcription by binding to DNA and forming cross-links. The drug is widely used in the treatment of gastrointestinal tumors. Here we used L-OHP as a positive control drug. After the treatment of KM and L-OHP at varying concentrations and measurement using the CCK-8 assay (Figure 1A), it showed significant inhibition of cell proliferation in a dose-dependent manner in HepG2 and Huh7 cells. CCK-8 assay results indicated that the half-maximal inhibitory concentration (IC50) of Koumine for HepG2 cells was 433.8 ± 19.1 μg/mL (≈1.42 mM), and for Huh7 cells was 394.2 ± 48.3 μg/mL (≈1.29 mM). The IC50 values of Oxaliplatin (L-OHP) were 4.789 ± 0.177 μg/mL (≈0.012 μM) for HepG2 cells and 10.01 ± 1.7 μg/mL (≈0.025 μM) for Huh7 cells. Further, clonogenic assays confirmed that both compounds markedly reduced the colony formation of liver cancer cells at these concentrations (Figure 1B). Additionally, The flow cytometry results showed that the apoptosis rates of HepG2 and Huh7 cells treated with KM are higher than those of the control group but lower than those of the L-OHP treatment group. Specifically, KM treatment induced approximately 10% apoptosis in HepG2 cells and around 7% apoptosis in Huh7 cells (Figure 2A). The results of the cell cycle experiment showed that KM treatment significantly reduced the proportion of the G2/M phase in HepG2 and Huh7 cells, indicating that the cells’ ability to enter mitosis was inhibited. Additionally, the proportion of the S phase in HepG2 cells increased, suggesting that KM treatment caused HepG2 cells to be arrested in the S phase (Figure 2B). Transwell and scratch assay analyses were conducted to evaluate the impact of KM and L-OHP on the migration of HCC cells. Results demonstrated a pronounced reduction in cell migration for both KM and L-OHP in a dose-dependent manner, as illustrated in Figure 3.

### 3.2. In Vivo Experiments Confirm the Anti-Tumor Effect of Koumine

**Figure 4:**
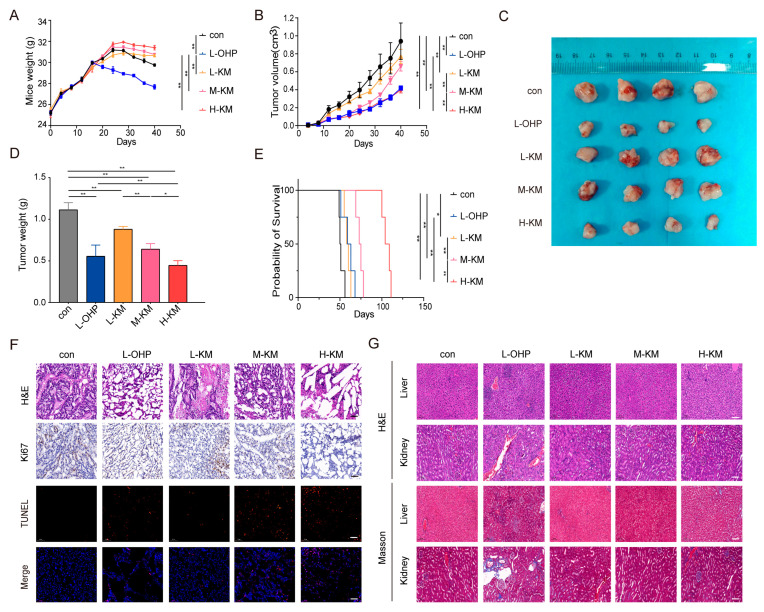
In vivo experimental results. (**A**) Mice weight in KM-treated and control groups. (**B**) The tumor growth curve in KM-treated and control groups. (**C**) Comparison of tumor size between different doses of KM-treated and control groups in the animal study. (**D**) Tumor weight in KM-treated and control groups. (**E**) Probability of Survival in KM-treated and control groups. (**F**) TUNEL and H&E and IHC images of Ki67 in tumor sections from KM-treated and control groups. Scale bar = 50 μm (**G**) Hematoxylin and eosin (H&E) and Masson’s trichrome staining of liver and kidney sections from KM-treated and control mice. Scale bar = 100 μm The data are shown as mean ± SD. * *p* < 0.05, ** *p* < 0.01.

To assess the in vivo therapeutic efficacy of KM, HepG2 cells were injected subcutaneously into Balb/c nude mice to create a tumor model. These mice were subsequently administered varying doses of KM and L-OHP as treatments for HCC. Figure 4A shows the changes in mouse body weight with different doses of KM and L-OHP treatment, with treatment days (0 to 40 days). The results indicate that the body weight of mice in all KM dose groups increased over time, with the high-dose group (H-KM) showing the most significant growth. Figure 4B illustrates the changes in tumor volume in different treatment groups, with treatment days (0 to 40 days). The H-KM group demonstrated the most significant inhibition of tumor growth, showing better efficacy than the L-OHP group. Figure 4C,D illustrated that, after 40 days of treatment, KM dose-dependently suppressed the cell growth of HCC, reducing tumor size and weight. Survival analysis showed a significant improvement in survival rates for the groups treated with KM compared to those receiving L-OHP, as depicted in Figure 4E.

Figure 4F shows the histological experimental results of tumor tissue. H&E staining shows the basic morphological structure of the tumor tissue. IHC staining demonstrated that KM dose-dependently decreased the expression of Ki67 and TUNEL assay demonstrated that KM dose-dependently promoted the apoptosis of HCC cells. H&E and Masson staining further showed that Koumine treatment did not cause liver and kidney tissue injury and fibrosis. However, L-OHP treatment caused liver and kidney collagen deposition (Figure 4G). These results indicated that there were no obvious signs of toxicity during Koumine treatment, emphasizing its therapeutic potential.Furthermore, after intraperitoneal injection of KM at doses of 8 mg/kg and 1 mg/kg in C57 mice, the mean concentration-time curve within 24 h is shown in Figure S1A,B. KM exhibited rapid absorption, short retention time in the body, and a large apparent volume of distribution.Blood biochemical analysis was performed to measure the levels of AST, ALT, ALP, and BUN in the peripheral blood of mice (as shown in Figure S1C–F). The treatment with L-OHP increased the expression of AST, ALT, and ALP in the blood, indicating some degree of liver damage in mice. However, no significant impact on BUN levels was observed. Different concentrations of koumine (KM) did not significantly affect these blood markers related to liver and kidney function (Figure S1C–F). In addition, tests on WBC, RBC, HCT, HBG, MCV, MCH, MCHC, and PLT revealed that L-OHP reduced red blood cell count, hemoglobin levels, and platelet concentration in mice, indicating some hematotoxicity. However, koumine at different concentrations showed no significant impact on these indicators, demonstrating good biological safety (Figure S1G–N). These results indicated that there were no obvious signs of toxicity during koumine treatment, emphasizing its therapeutic potential.

### 3.3. Selection of Compound-Disease-Related Targets via Network Pharmacology

**Figure 5:**
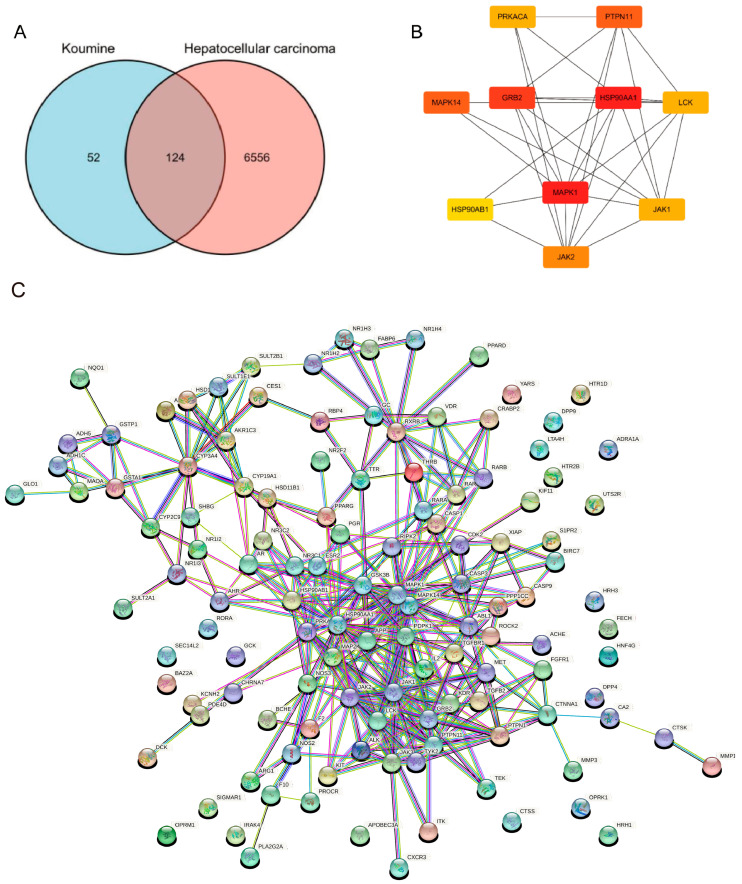
Selection of HCC-related targets. (**A**) Venn diagram of Koumine targets and HCC targets. (**B**) The core targets for the anti-HCC of Koumine. (**C**) Drug-component-disease-target network diagram.

**Figure 6:**
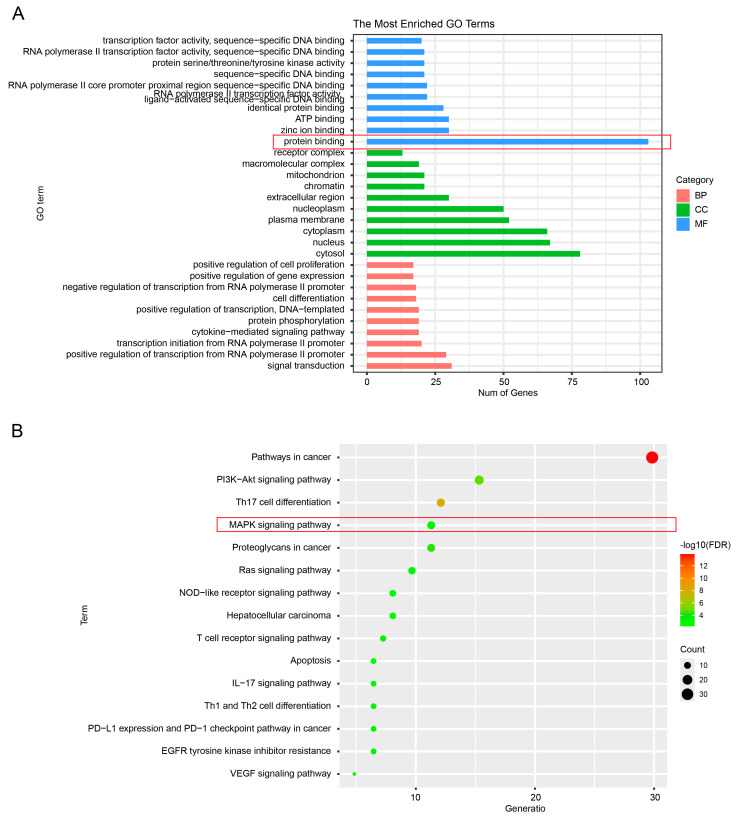
GO and KEGG enrichment analysis. (**A**) The top 30 most enriched GO categories and pathways were calculated and plotted. (**B**) Top 15 KEGG pathways. The red boxes highlight the ‘protein binding’ term in the GO analysis and the ‘MAPK signaling pathway’ in the KEGG analysis.

All of Koumine’s foundational data were gathered from the PubChem database (see Table 1). An analysis employing a Venn diagram revealed 124 overlapping targets between 176 potential Koumine targets and 6680 HCC targets, depicted in Figure 5A. Figure 5B illustrates the core targets for the anti-HCC of Koumine. Figure 5C illustrates the Protein–Protein Interaction (PPI) network involved in Koumine’s activity against HCC, featuring 24 nodes and 338 edges, demonstrating an average connectivity of 5.45 per node. Utilizing the CytoHubba plugin, we identified the top 10 pivotal targets in the network according to PPI degree values. These key targets—PRKACA, PTPN11, P38, GRB2, HSP90AA1, LCK, HSP90AB1, JAK1, and JAK2—are believed to be instrumental in mediating Koumine’s therapeutic impact on HCC. The GO Pathway and KEGG Enrichment Analysis of the 124 potential anti-HCC targets of Koumine, depicted in Figure 6, reveals its involvement in diverse biological processes, notably signal transduction, positive regulation of RNA polymerase II promoter transcription, cytokine-mediated signaling, and protein phosphorylation. Such multifaceted involvement underscores the comprehensive anti-HCC mechanism of Koumine. Moreover, its molecular function enrichment—primarily in protein, zinc ion, and ATP binding, along with RNA polymerase II transcription factor activity and sequence-specific DNA binding influenced by ligand interaction—further elucidates its potential therapeutic roles.

The anti-HCC effect of Koumine is associated with its modulation of multiple signaling pathways, including cancer-related pathways, PI3K-Akt, MAPK, Ras, apoptosis, IL-17, PD-1/PD-L1 checkpoint, EGFR tyrosine kinase inhibitor resistance, and VEGF signaling, as well as processes such as Th17/Th1/Th2 cell differentiation, extracellular matrix interactions in cancer, NOD-like receptor signaling, and hepatocellular carcinoma-specific mechanisms. These findings highlight the multi-faceted therapeutic potential of Koumine against HCC.

**Figure 7:**
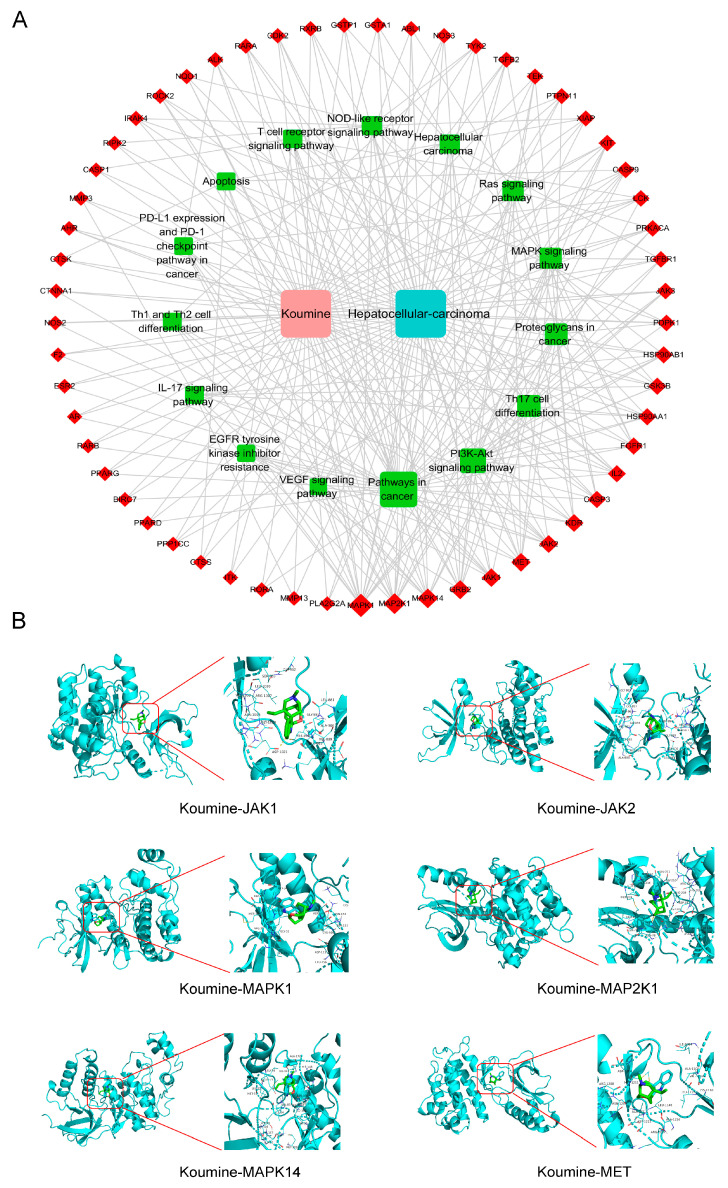
Molecular docking. (**A**) Drug-component-disease-target network. (**B**) Virtual docking of the binding of JAK1, JAK2, MAPK1, MAP2K1, MAPK14 (P38), and MET with Koumine shown as 3D diagrams.

Using Cytoscape, we constructed a CTPD interaction network (Figure 7A), with node size scaled to connectivity. Key targets—MAPK1, MAP2K1, P38, GRB2, JAK1, and MET—were identified as central across multiple pathways, underscoring their pivotal role in the anti-HCC mechanism of Koumine. This network visualization further supports that Koumine acts through a complex, multi-target signaling network against HCC.

Molecular docking studies indicated that Koumine exhibits significant binding affinity to MAPK1, MAP2K1, P38, JAK1, JAK2, and MET, with binding energies below −5.0 kcal/mol (Table 3). Notably, Koumine showed the strongest interaction with JAK2, achieving a binding energy of −8.53 kcal/mol. Additionally, Koumine formed stable hydrogen bonds with JAK1 (ASP1021) and MAP2K1 (LYS97). However, due to the lack of a ligand-bound GRB2 structure in the PDB database, docking analysis for this target was not performed. Interaction details between Koumine and key targets are illustrated in Figure 7B.

### 3.4. Selection of Compound-Disease-Related Targets via Proteomics

**Figure 8:**
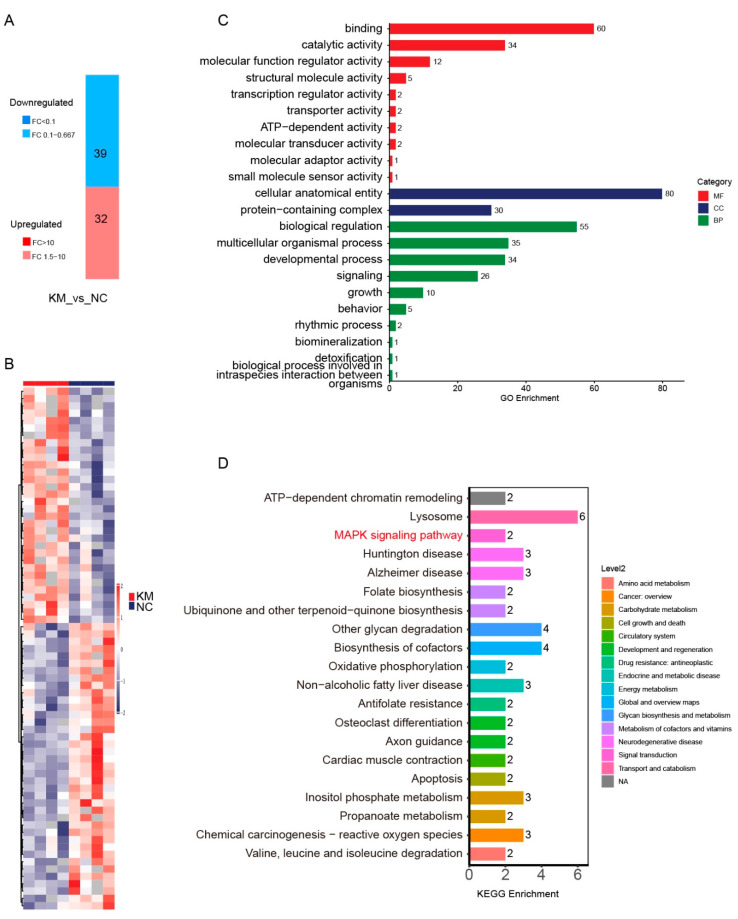
Analysis of differentially expressed proteins in tumor samples. (**A**) Number of differentially expressed proteins; (**B**) Heatmap of cluster analysis of differentially expressed proteins in tumors treated with or without Koumine. The GO pathway (**C**) enrichment (MF: Molecular Function; CC: Cellular Component; BP: Biological Process) and KEGG enrichment (**D**) is shown.

Based on proteomic analysis of hepatocellular tumor tissues from mice, the Koumine-treated group showed upregulation of 32 proteins and downregulation of 39 proteins compared to controls (Figure 8A). A heatmap clustered and displayed the expression patterns of these proteins (Figure 8B). GO enrichment analysis highlighted terms including catalytic activity, molecular function regulator, cellular anatomical entity, protein-containing complex, and biological regulation (Figure 8C). KEGG pathway analysis further identified significant enrichment in the MAPK signaling pathway, consistent with earlier network pharmacology results (Figure 8D).

### 3.5. Impact of Koumine on P38 Pathway in HCC Cells

**Figure 9:**
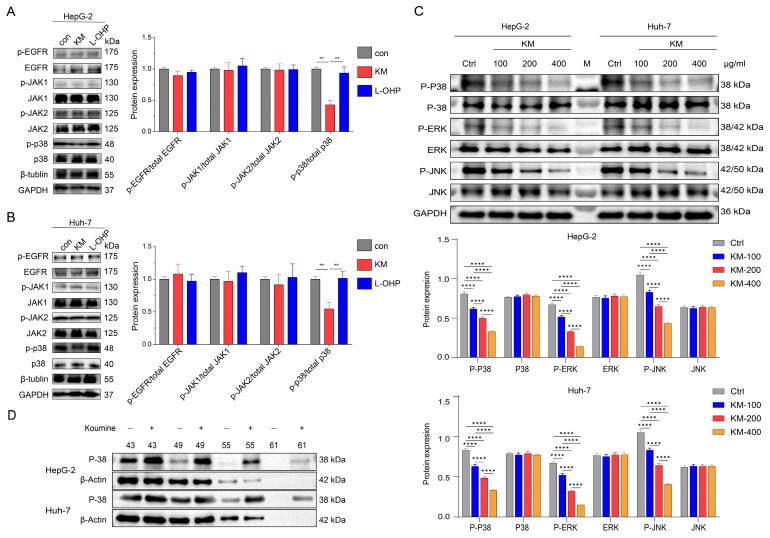
Impact of Koumine on protein expression in HCC Cells. (**A**,**B**) Western blot results of p-EGFR, EGFR, p-JAK1, JAK1, p-JAK2, JAK2, p-P38 and P38 protein expression in KM-treated and control HepG2, Huh7 cells, showing a significant down-regulation of p-p38 protein in the KM-treated group. (**C**) Western blot analysis of P-P38, P38, P-ERK, ERK, P-JNK and JNK protein levels in HepG2 and Huh7 cells with different concentrations of KM. (**D**) Western blot analysis of P38 protein levels in HepG2 and Huh7 cells with or without KM in different temperatures. Data represent three independent experiments. Error bars, mean ± SD. ** *p* < 0.01, **** *p* < 0.0001.

Immunoblotting analysis in Koumine-treated HCC cells showed that the protein expression of p-P38 was notably reduced compared to controls in both HepG2 and Huh7 cells (Figure 9A,B). Further dose–response studies indicated that Koumine treatment downregulated p-P38, p-ERK, and p-JNK in a concentration-dependent manner (Figure 9C). These results align with network pharmacology predictions, suggesting that Koumine’s anti-HCC effect is partly mediated by inhibiting MAPK pathway phosphorylation. Moreover, CETSA results showed enhanced thermal stability of P38 in Koumine-treated cells, indicating direct binding between Koumine and P38 (Figure 9D).

## 4. Discussion

**Figure 10:**
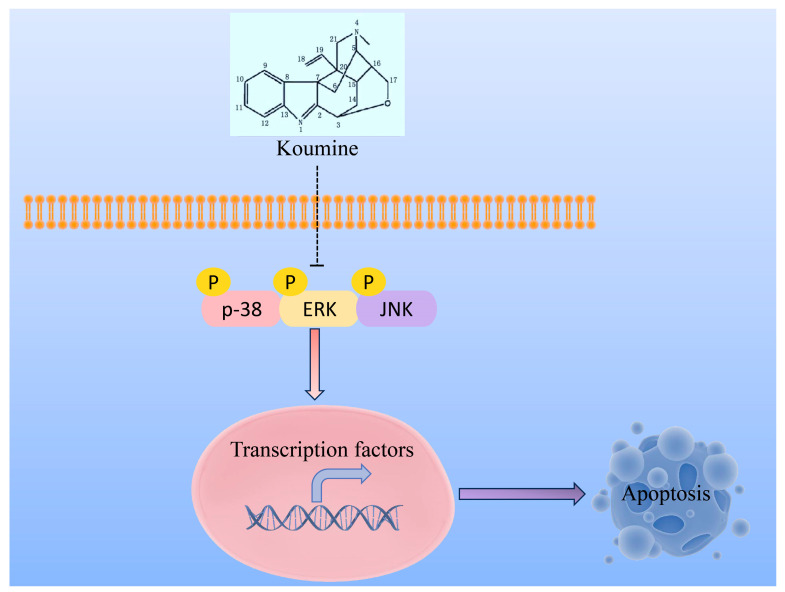
Koumine promoted the apoptosis of HCC cells by inhibiting the phosphorylation of P38, ERK, and JNK.

Our study has demonstrated that Koumine effectively inhibits the proliferation and migration of HCC cells by specifically targeting P38 (Figure 10). Treatment with Koumine suppressed proliferation and migration and induced cell cycle arrest in HCC cells. Despite significant advancements in cancer therapy over the past few decades, treatment options for patients with HCC remain severely limited, highlighting the urgent need for novel therapeutic interventions. Our findings suggest that Koumine may represent a promising therapeutic avenue for HCC.

The clinical treatment of HCC continues to confront significant challenges, including prevention difficulty, late diagnosis, and limited therapeutic targets. Despite the potential of targeted combination immunotherapy, it suffers from a low overall response rate and considerable variability of the patient-reported outcomes. Consequently, the prognosis for patients with advanced liver cancer remains bleak [2]. Traditional Chinese Medicine (TCM), with its rich heritage, plays an indispensable role across different stages of liver cancer management. And it effectively complements Western medical approaches during the preoperative, postoperative, follow-up, and palliative care phases [16].

Koumine, extracted from the traditional Chinese medicinal herb *Gelsemium elegans* Benth, demonstrates various pharmacological and biological effects. It has been previously shown to be effective against several cancers, including colorectal, lung, and gastric cancer [13,14]. In this study, we explored the potential anti-HCC properties of Koumine. Our findings further confirm its significant efficacy against HCC, supporting our prior assumptions. Integrating network pharmacology, molecular docking, and experimental validation, we have particularly elucidated the action mechanisms of Koumine, highlighting the potential of Chinese herbal medicine and its compounds [17,18]. The methodological robustness of this integrative approach is further supported by recent studies demonstrating the feasibility and value of combining network pharmacology with proteomics for target deconvolution in natural product research [19,20]. Our network pharmacology analysis also found 124 potential targets between Koumine and HCC, with 10 core proteins being PRKACA, PTPN11, P38, GRB2, HSP90AA1, LCK, HSP90AB1, JAK1, and JAK2. GO and KEGG analyses further highlighted Koumine’s impact on diverse biological processes, including protein, zinc ion, and ATP binding; RNA polymerase II transcription factor activity; and ligand-activated sequence-specific DNA binding. Importantly, KEGG analysis emphasized the role of the MAPK signaling pathway in HCC. A compound-target-pathway-disease network was also established, indicating the potential targeting of key proteins like MAP2K1, P38, GRB2, JAK1, and MET of Koumine in combating HCC. Comparing the predicted target profile of Koumine with those of other plant-derived alkaloids reveals both shared and distinct pathway engagements, underscoring the unique polypharmacology of this compound [19].

The decision to prioritize the MAPK and JAK pathways for experimental validation was guided by the convergence of computational and experimental evidence. KEGG enrichment analysis of the 124 network pharmacology targets identified the MAPK signaling cascade as a prominently enriched pathway (Figure 6B). Concurrently, PPI network topological analysis revealed JAK1 and JAK2 as core hub targets with high degree centrality (Figure 5B). Importantly, proteomic profiling of Koumine-treated tumor tissues independently corroborated the significant enrichment of the MAPK pathway (Figure 8D), thereby providing a robust, data-driven rationale for focusing subsequent mechanistic investigations on the MAPK and JAK axes.

The P38 MAPK cascade, distinct from the Ras-Raf-MEK-ERK pathway, is activated by cellular stress and inflammatory cytokines through upstream kinases such as MKK3/6, leading to the phosphorylation of P38. Research by Huang et al. found that Koumine can induce apoptosis and halt the cell cycle in cancer cells by blocking the ROS-dependent NF-κB signaling pathway [21]. Furthermore, studies by Yuan et al. have demonstrated that Koumine reduces the production of pro-inflammatory factors in mouse macrophages by inhibiting the phosphorylation of ERK/P38 MAPK and the activity of the NF-κB pathway [22]. Lee et al. [23] explored how Koumine modulates NF-κB and ERK/P38 MAPK signaling pathways, thereby inhibiting the proliferation of HCC cells and enhancing apoptosis. The MAPK/ERK pathway, essential for cell survival and proliferation, becomes aberrantly activated in tumor carcinogenesis [24]. Given this evidence and the identified targets, the anti-HCC effect of Koumine appears to be more linked to targeting P38.

Through in vitro studies, we investigated the impact of Koumine on apoptosis and the migratory behavior of HCC cells. The results indicate that Koumine effectively suppresses HCC cell proliferation, and enhances apoptosis, and this apoptotic activity would strengthen with increasing concentrations. At optimal concentrations, Koumine triggers apoptosis at the G0/G1 and S phases of the cell cycle, underscoring its anticancer efficacy. The observed S-phase arrest in HepG2 cells suggests a potential involvement of cell cycle regulatory pathways beyond the MAPK axis. Recent studies have implicated the CDK6/E2F1 signaling axis as a critical node in HCC cell cycle progression and immune evasion, warranting future exploration of whether Koumine modulates this pathway and its associated immunological consequences [25]. Furthermore, it significantly reduces the migratory and invasive capabilities of HCC cells, corroborating previous research on the anticancer properties of Koumine. By integrating our findings with prior network pharmacology insights and the confirmation via Western blot analysis, we established a novel theoretical basis for employing Koumine in HCC treatment.

In vivo experiments, between the Koumine-treated group and the control group, we observed significant differences in tumor weight, revealing a dose-dependent decrease in tumor weight accompanied by the dosage increase of Koumine. Unlike mice treated with Oxaliplatin (the positive control), which experienced weight loss, reduced activity, and deteriorated mental states, Koumine-treated mice maintained stable body weights, exhibited healthy mental conditions, and showed improved responsiveness, suggesting minimal side effects. Oxaliplatin, used here as a positive control, is a first-line treatment for HCC [26]. Compared to Oxaliplatin, Koumine offers benefits such as engaging multiple signaling pathways with fewer adverse effects, suggesting a promising choice for HCC treatment. CCK-8 assay results indicated that the half-maximal inhibitory concentration (IC50) of Koumine for HepG2 cells was 433.8 ± 19.1 μg/mL (≈1.42 μM), and for Huh7 cells was 394.2 ± 48.3 μg/mL (≈1.29 μM). In contrast, the IC50 values of Oxaliplatin (L-OHP) were 4.789 ± 0.177 μg/mL (≈0.012 μM) for HepG2 cells and 10.01 ± 1.7 μg/mL (≈0.025 μM) for Huh7 cells. These data indicate that the effective concentration of Koumine is substantially higher than that of the clinical chemotherapeutic agent Oxaliplatin, pointing to its relatively lower in vitro potency against HCC and suggesting that structural optimization or combination strategies may be necessary to enhance its therapeutic applicability. Due to the promising potential of Koumine, refinement of these compounds to enhance their pharmacological efficacy, minimize side effects, and improve pharmacokinetics and drug-like qualities is warranted. Subsequent chemical modifications aim to optimize these compounds into viable drug candidates [27]. Many modern drugs originated from plant-derived substances with good clinical efficacy, including quinine, paclitaxel, and artemisinin, etc. [28,29,30]. Plant alkaloids, animal toxins, and microbial metabolites have served as foundations for developing new therapeutics, exemplified by statins such as lovastatin and simvastatin [31]. Given its abundance and low toxicity, Koumine from Gelsemium elegans Benth holds potential for drug development.

With the progression of the recent advancements in HCC treatment, people have positioned drugs such as cisplatin and sorafenib as the cornerstone of first-line therapy. These agents primarily exert their therapeutic effects by inducing cell death and provoking inflammatory responses to suppress tumor growth [32]. Extensive research underscores the critical function of reactive oxygen species (ROS) in influencing the mitogen-activated protein kinase (MAPK) signaling pathway. This pathway is vital for signal transduction and plays a central role in coordinating numerous cellular activities, such as proliferation, differentiation, cell cycle arrest, survival, and apoptosis [33]. The MAPK family includes crucial elements like extracellular signal-regulated kinases (ERK), c-Jun N-terminal kinases (JNK), and p38, which are instrumental in controlling tumor cell behavior, encompassing proliferation, apoptosis, and anti-inflammatory actions. Consequently, the therapeutic impact on HCC is likely mediated by activation of the MAPK pathway [34]. Furthermore, studies have revealed that MAPK phosphorylation levels in HCC tissues are significantly elevated, sevenfold higher than those in surrounding non-tumorous tissues [35].

In our research, we utilized immunoblotting to analyze the expression levels of P38 and its phosphorylated form, p-P38. The findings indicate that Koumine significantly reduces the levels and phosphorylation activity of P38, suggesting possible mechanisms for its anti-HCC activity. In addition, the effect of different concentrations of Koumine on P38 and p-P38 expression was investigated, our study found that the treatment of Koumine resulted in a notable reduction in the protein expression of p-P38, p-ERK, and p-JNK in a dose-dependent manner. The result of the CETSA assay confirmed the binding of Koumine to P38. These findings confirm that Koumine suppresses the activation of the MAPK signaling cascade in HCC cells.

Despite these promising findings, several limitations should be acknowledged. First, the in vivo efficacy of Koumine was only assessed in subcutaneous xenograft models, which may not fully recapitulate the tumor microenvironment of orthotopic or spontaneous HCC. Second, while we identified P38 as a direct binding target via CETSA, the exact binding pocket and downstream structural consequences require further crystallographic or mutagenesis studies. Third, the relatively high IC_50_ of Koumine compared to conventional chemotherapeutics suggests that structural optimization or combination regimens may be necessary to enhance its clinical applicability. Fourth, although our histological examination of liver and kidney tissues revealed no overt signs of fibrosis or organ damage following Koumine treatment, comprehensive safety evaluations—including the assessment of hepatic fibrosis biomarkers and long-term toxicity parameters—remain essential for future translational development [36]. Future studies will focus on elucidating the structure-activity relationship of Koumine derivatives, evaluating its pharmacokinetic profiles in larger animal models, and exploring its synergistic potential with existing targeted therapies or immunotherapies for HCC. Furthermore, given the emerging evidence that natural alkaloids can modulate drug resistance mechanisms across multiple cancer types, investigating whether Koumine can sensitize HCC cells to conventional chemotherapeutics or overcome acquired resistance represents a promising direction for future research [37].

Our findings lay the groundwork for deeper exploration into the modulation of signaling pathways of Koumine against HCC. Based on this research and its preliminary results, Koumine has the potential to become a promising candidate for HCC treatment. However, more extensive research is required to further elucidate its pharmacological actions and to assess its clinical utility comprehensively.

## 5. Conclusions

Network pharmacology analysis indicates that Koumine exerts anti-HCC effects via a multi-target, multi-pathway network. In vitro experiments demonstrate that Koumine suppresses the proliferation, migration, and invasion of HCC cells, while also inducing apoptosis and arresting the cell cycle at the G0/G1 and S phases. Mechanistic studies, integrating network pharmacology and immunoblotting, suggest that the anti-HCC activity of Koumine is associated with inhibition of the P38 signaling pathway. In vivo experiments further confirm that Koumine reduces tumor growth and prolongs survival in a murine HCC model. These findings collectively support Koumine as a promising therapeutic candidate for HCC with the potential to improve clinical outcomes.

## Figures and Tables

**Table 1:** The basic parameters of Koumine.

Parameter Name	Parameter Value
Compound	Koumine
PubChem CID	102004413
Cas	1358-76-5
Structural Formula	C20H22N2O
Molecular Weight (g/mol)	306.4
Canonical SMILES	CN1CC2(C3CC4C5=NC6=CC=CC=C6C52CC1C3CO4)C=C
Constitutional Formula	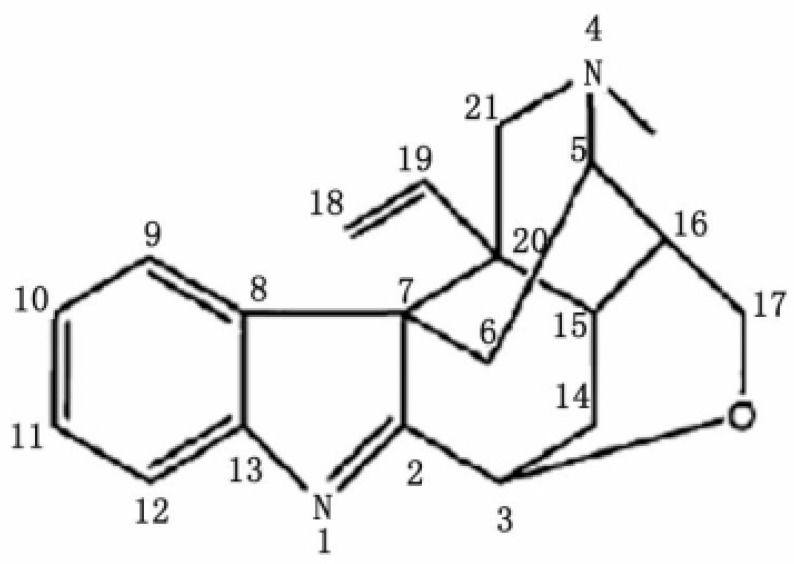

**Table 2:** The basic parameters of Koumine based on HPLC.

Parameter Name	Parameter Value
Instrument	Waters-HPLC
Column	C18, 4.6 × 250 nm, 5 μm
Detection Mode:	260 nm
Column Temperature	35 °C
Flow Rate	1.0 mL/min
Sample dissolution Mobile Phase Gradientelution	60% methanol in water A: Acetonitrile B: 0.1% Triethylamine phosphate in water A: 10%–30%, 0–15 min
ChemoPprofile	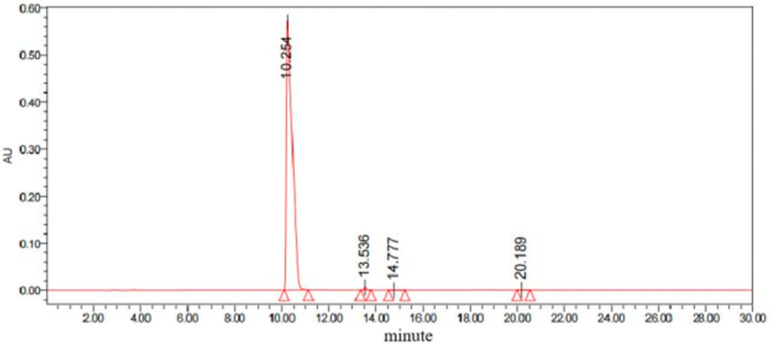

**Table 3:** The results of Molecular docking.

Target	PDB: ID	Ligand	Affinity (KJ/mol)
MAPK1	5ik4	NA	−7.83 ± 0.02
MAP2K1	7b94	LYS′97	−6.97 ± 0.01
MAPK14	6sfi	NA	−7.23 ± 1.57
JAK1	4ehz	ASP′1021	−7.56 ± 0.01
JAK2	4d1s	NA	−8.53 ± 0.02
MET	3dkg	NA	−6.38 ± 0.00

## Data Availability

The authors confirm that the data supporting the findings of this study are available within the article.

## Supplementary Materials

The following supporting information can be downloaded at: https://www.mdpi.com/article/10.3390/biomedicines14061250/s1, Supplementary File S1: Putative Targets of Koumine Predicted by TargetNet, File S2: Candidate Targets of Koumine Identified Using SwissTargetPrediction, File S3: Putative Targets of Koumine Predicted by PharmMapper Analysis, Figure S1: Pharmacokinetic profiles of KM in mice and its effects on liver/kidney function and hematological parameters.

## Abbreviations

ANOVA, One-way analysis of variance; ATCC, American Type Culture Collection; BCA, bicinchoninic acid; Bcl-2, B-cell lymphoma/leukemia-2; BP, biological process; BSA, bovine serum albumin; CCK-8, Cell counting kit 8; CETSA, Cellular thermal shift assay. CytC, cytochrome oxidase; ECL, enhanced chemiluminescence; EGFR, epidermal growth factor receptor; ERK, extracellular-signal regulated protein kinase; Fc, fold change values; GEB, *Gelsemium elegans* Benth; GO, Gene Ontology; HCC, hepatocellular carcinoma; IC50, the half maximal inhibitory concentration; JAK1, Januskinase1; JAK2, Januskinase2; JNK, c-Jun N-terminal kinases; KEGG, Kyoto Encyclopedia of Genes and Genomes; KM, Koumine; LFQ, label-free quantification; L-OHP, oxaliplatin; MAPK1, mitogen-activated protein kinase 1; MAPK14,mitogen-activated protein kinase 14; PBS, phosphate-buffered saline; PDB, Protein Data Bank; PPI, Protein–protein interaction; PVDF, polyvinylidene fluoride; TCM, Traditional Chinese Medicine; TUNEL, terminal deoxynucleotidyl transferase dUTP nick end labeling.
