# Supplementary figures and images for: Koumine’s Therapeutic Impact on Hepatocellular Carcinoma: A Combined Network Pharmacology and Experimental Study
Source: Biomedicines. 2026 May 30;14(6):1250. doi: 10.3390/biomedicines14061250 (PMC13296294; doi:10.3390/biomedicines14061250)

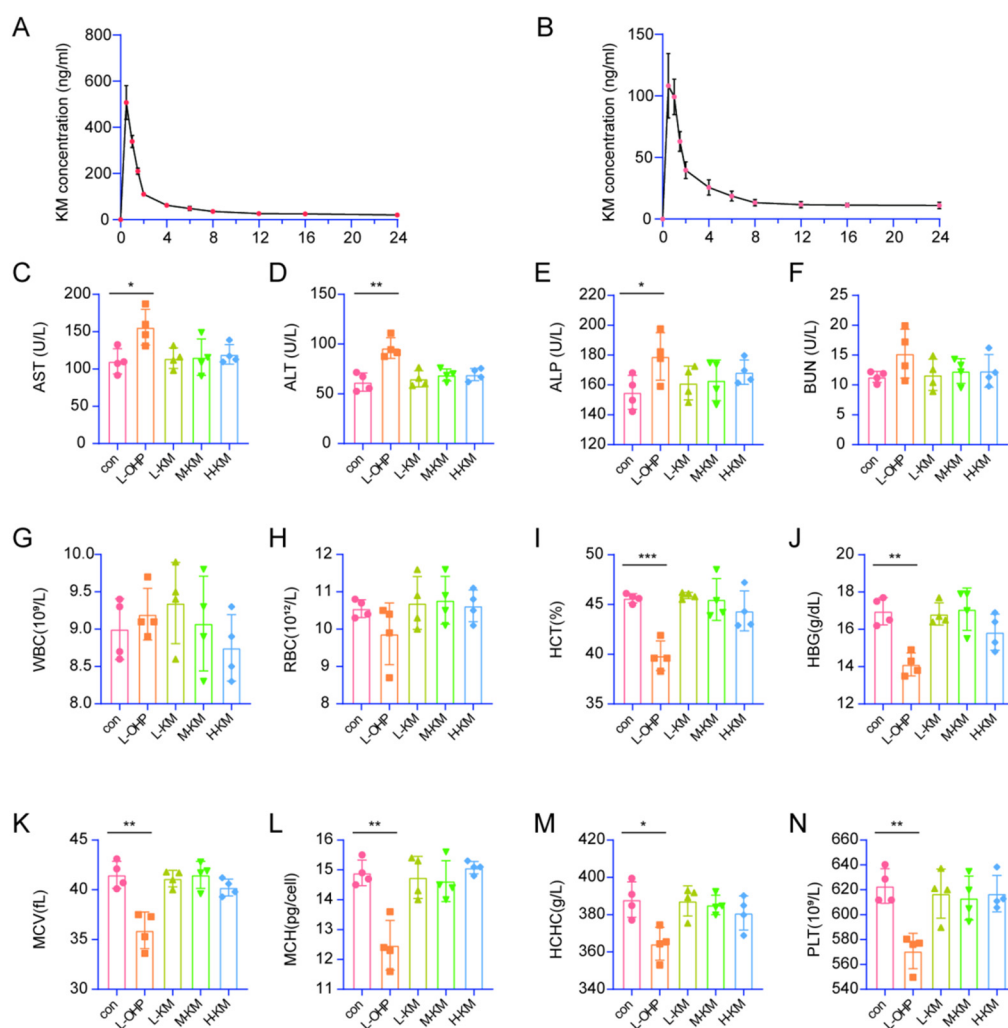

Supplement: Supplementary file 1 [file biomedicines-14-01250-s001.zip › Supplementary Materials Figure S1.pdf]
